# Redox Changes Induced by General Anesthesia in Critically Ill Patients with Multiple Traumas

**DOI:** 10.1155/2015/238586

**Published:** 2015-11-26

**Authors:** Marius Papurica, Alexandru Florin Rogobete, Dorel Sandesc, Raluca Dumache, Radu Nartita, Mirela Sarandan, Alina Carmen Cradigati, Loredana Luca, Corina Vernic, Ovidiu Horea Bedreag

**Affiliations:** ^1^Clinic of Anesthesia and Intensive Care, Emergency County Hospital “Pius Brinzeu”, 300736 Timisoara, Romania; ^2^Faculty of Medicine, “Victor Babes” University of Medicine and Pharmacy, 300041 Timisoara, Romania; ^3^Faculty of Chemistry, Biology, Geography, West University of Timisoara, 300115 Timisoara, Romania; ^4^Clinic of Anesthesia and Intensive Care “Casa Austria”, Emergency County Hospital “Pius Brinzeu”, 300736 Timisoara, Romania

## Abstract

The critically ill polytrauma patient is a constant challenge for the trauma team due to the complexity of the complications presented. Intense inflammatory response and infections, as well as multiple organ dysfunctions, significantly increase the rate of morbidity and mortality in these patients. Moreover, due to the physiological and biochemical imbalances present in this type of patients, the bioproduction of free radicals is significantly accelerated, thus installing the oxidative stress. In the therapeutic management of such patients, multiple surgical interventions are required and therefore they are being subjected to repeated general anesthesia. In this paper, we want to present the pathophysiological implications of oxidative stress in critically ill patients with multiple traumas and the implications of general anesthesia on the redox mechanisms of the cell. We also want to summarize the antioxidant treatments able to reduce the intensity of oxidative stress by modulating the biochemical activity of some cellular mechanisms.

## 1. Introduction

The critically ill polytrauma patient requires a multidisciplinary management due to the complexity of injuries [1]. The most common injuries are represented by traumatic brain injury, spinal cord injury, traumatic injuries of the lung parenchyma, abdominal traumas, traumatic injuries of the pelvis and of extremities, and a number of phenomena that usually accompany the multiple traumas, such as hemorrhagic shock or hypothermia [2–4]. Moreover, a series of secondary injuries are installed after the primary injuries such as systemic inflammatory response syndrome (SIRS), sepsis, and eventually multiple organ dysfunction syndrome (MODS) [5, 6]. A high percentage of patients with multiple traumas are requiring emergency surgery at the admission time or several times during the length of stay in the intensive care unit (ICU). General anesthesia is required in order to ensure the need for the surgical interventions. Numerous studies have highlighted a number of molecular changes induced by anesthetic substances, regarding the redox balance [7, 8]. Due to hypermetabolism and due to severe generalized inflammations, the critically ill polytrauma patient shows a tropism for the aggressive production of free radicals (FR) ultimately responsible for the installation of the phenomenon called oxidative stress (OS) [9, 10].

In this paper, we want to present the pathophysiological implications of OS in the critically ill patient with multiple traumas, as well as its modulation by general anesthesia. We also want to summarize the existing antioxidant methods currently used to minimize OS in this type of patients.

## 2. Biochemical and Pathophysiological Aspects of Oxidative Stress

**Figure 1:**
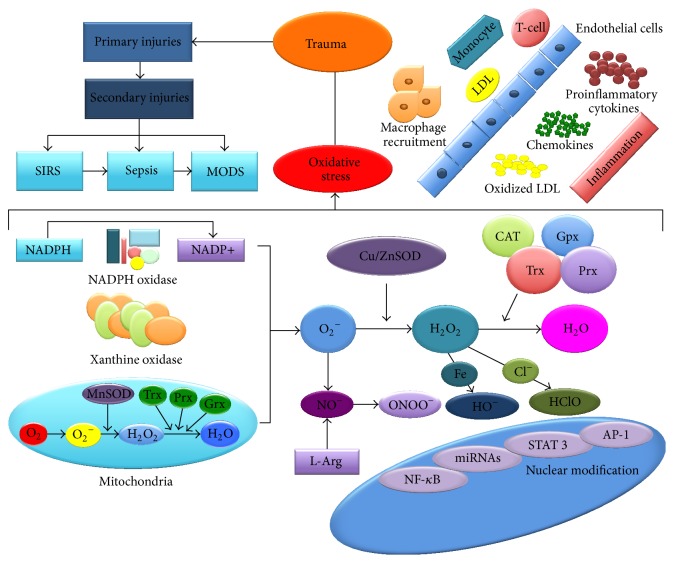
Schematic representation of oxidative stress in the critically ill patient with multiple traumas. Primary trauma induces a series of secondary injuries due to the biological and biochemical imbalances. The first side effect installed is SIRS, followed by sepsis, and finally by MODS. The inflammations generated by the action of the polymorphonuclear cells, as well as by hypermetabolism, maintain and enhance the oxidative stress. Mitochondria are significantly affected thereby producing significant amounts of superoxide anion. Free radicals produced at the cellular level are neutralized by the number of antioxidant enzyme systems, such as SOD, CAT, Trx, Gpx, and Prx [11, 12].

Due to the cell redox activity in physiological conditions, a series of highly reactive species called FR are being produced. The most important FR classes are represented by reactive oxygen species (ROS), reactive nitrogen species (RNS), and reactive lipid species (RLS) [27–29]. The cell redox potential is influenced mostly by nicotinamide adenine dinucleotide phosphate (NADPH) oxidases, xanthine oxidase, cytochrome P450 isoenzymes, cyclooxygenase, endothelial NO synthase, lipoxygenase, and hemeoxygenase [30–32] (Figure 1).

FR oxidative activity is kept under control by endogenous antioxidant systems, represented by a number of antioxidant enzymes and some vitamins. The most potent endogenous antioxidant systems are represented by antioxidant enzymes, such as glutathione (GSH), thioredoxin (Trx), glutaredoxins (Grx), superoxide dismutase (SOD), catalase (CAT), paraoxonase (PON), and peroxiredoxins (Prx) [20, 22, 33, 34]. In Table 1 the most important endogenous antioxidant systems are summarized.

In the critically ill polytrauma patient, the antioxidant/oxidant ratio is disrupted, thus the oxidative stress (OS) being installed (Figure 1). Along with increasing OS intensity, a number of cellular biological systems are disrupted because of DNA and protein distortion or because of lipoprotein membrane oxidation, resulting in the blockage of the cellular biochemical activity. The accelerated bioproduction of FR is involved in the DNA modulation reactions, the specific biomarkers in this regard being 8-hydroxydeoxyguanosine (8-OHdG) [35]. For lipid oxidation, malondialdehyde (MDA), 4-hydroxynonenal (4-HNE), and isoprostanes (IsoPS) were identified and used as biomarkers [36]. Also, for protein oxidation, the carbonyl group and nitrotyrosine have been identified [37–40].

In the case of severe traumas, shortly after the traumatic injury, the systemic inflammatory response syndrome (SIRS) is installed [5]. Specialized studies have demonstrated that OS is responsible for the amplification of inflammations. From a genetic point of view, OS modulates the inflammation by regulating the activity of some transcription factors, such as nuclear factor-kappa B (NF-*κ*B), signal transducer and activator of transcription 3 (STAT 3), hypoxia inducible factor 1-alpha (HIF-1-alpha), and activator protein-1 (AP-1) [32, 41–44]. By stimulating the activity of these factors, the proinflammatory mediators are being excessively secreted. They are responsible for the dissemination and the enhancement of the inflammatory response. NF-*κ*B is considered to be the most important transcription factor regarding the inflammatory response, being responsible for the excessive synthesis of some proinflammatory cytokines such as interleukin 1 (IL-1), interleukin 6 (IL-6), interleukin 8 (IL-8), and tumor necrosis factor-alpha (TNF-alpha) [45–47]. From a structural point of view, NF-*κ*B is composed of two subunits, p50 and p65. This is physiologically found in the cytoplasm, forming a complex with an inhibitory protein (IkB). Along with the cell stimulation, IkB is phosphorylated in two residual groups of serine. OS favors the activation of the cell surface and therefore is modulating the activity of NF-*κ*B in the nucleus [27, 48, 49].

Specialized studies show a significant increase in morbidity and mortality at the polytrauma patients that have suffered from complications due to severe inflammations. One of the organs most affected in this regard is the lung. Thoracic trauma, according to the trauma reports, is one of the most prevalent in this type of patients [50]. Severe injuries of the pulmonary parenchyma are associated with an increased morbidity and mortality due to the primary injury as well as to the secondary complications. Among them, the most severe injuries are represented by the acute lung injury (ALI) and by the acute respiratory distress syndrome (ARDS) [51, 52]. In these cases, not only because of the excessive production of proinflammatory molecules, but also because of the activation of the polymorphonuclear cells, OS is significantly intensified [53]. Another system significantly affected, especially in patients with hemorrhagic shock, is the microvascular system. This system is composed of small blood vessels with a diameter smaller than 100 microns, which are responsible for tissue oxygenation, nutrition, and immune modulation. Hemorrhagic shock, infections, and inflammations are leading to microcirculatory dysfunction. Induced endothelial dysfunction also leads to the excessive production of FR and therefore to the intensification and generalization of OS [54–56].

## 3. Chemical Aspects of General Anesthetics

**Figure 2:**
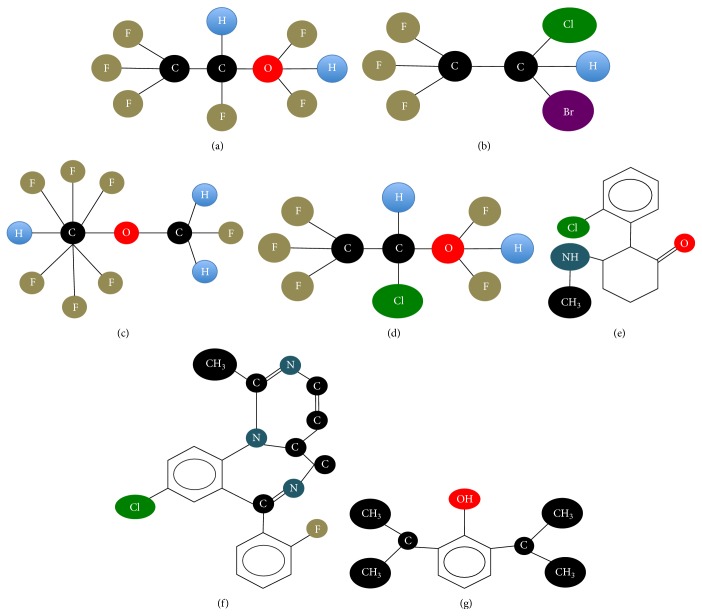
The chemical structure of (a) desflurane, (b) halothane, (c) isoflurane, (d) sevoflurane, (e) ketamine, (f) midazolam, and (g) propofol.

The most commonly used substances in general anesthesia are volatile anesthetics, such as desflurane, halothane, isoflurane, and sevoflurane. Other anesthetics substances that are not volatile are also used in the present such as ketamine, midazolam, and propofol. Volatile anesthetics are from a chemical point of view halogenated substances. According to the International Union of Pure and Applied Chemistry (IUPAC, http://www.iupac.org/), desflurane is fluorinated ether with molecular formula 2-(difluoromethoxy)-1,1,1,2-tetrafluoroethane. Halothane is a halogenated hydrocarbon anesthetic with molecular formula 2-bromo-2-chloro-1,1,1-trifluoroethane. Isoflurane is a fluorinated ether with molecular formula 2-chloro-2-(difluoromethoxy)-1,1,1-trifluoroethane and sevoflurane is a fluorinated isopropyl ether with molecular formula 1,1,1,3,3,3-hexafluoro-2-(fluoromethoxy)propane [57–59]. Propofol belongs to the class of hypnotic alkylphenol derivatives, with molecular formula 2,6-di(propan-2-yl)phenol. Ketamine is a cyclohexanone derivative with anesthetic properties and with molecular formula 2-(2-chlorophenyl)-2-(methylamino)cyclohexan-1-one. Midazolam is a benzodiazepine with molecular formula 8-chloro-6-(2-fluorophenyl)-1-methyl-4H-imidazo[1,5-a][1,4]benzodiazepine (Figure 2) [60–62].

## 4. Molecular Changes Induced by General Anesthesia

**Figure 3:**
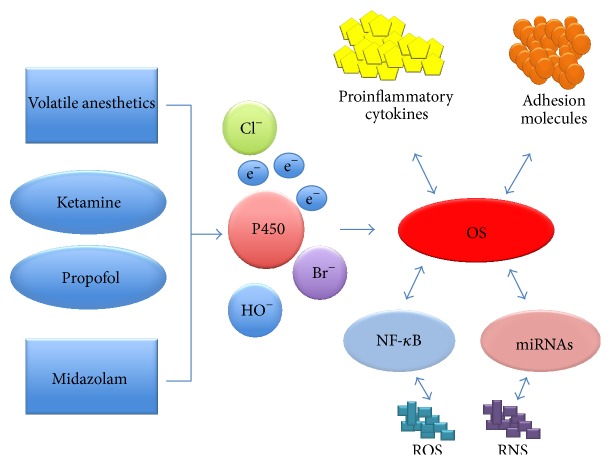
The implications of general anesthetics in the cellular redox activity.

Regarding the molecular changes induced by general anesthetics, there are a series of studies that highlight the implications of these substances in the cellular metabolisms. Morio et al. report in a study on biotransformation reactions of general anesthetics several FR species that resulted from specific metabolic reactions. The majority of general anesthetics are degraded by enzymatic systems such as cytochrome P450 located in cytoplasm and mitochondria [62–64]. The enzymatic reactions of degradation of anesthetic substances can be grouped in two phases, phase I of oxidation or reduction and phase II of conjugation or synthesis. In the study conducted by them on the biotransformation of halothane, they have identified a series of metabolic compounds responsible for enhancing and maintaining the OS. Chlorodifluoroethylene and chlorotrifluoromethane were found in the exhaled air and trifluoroacetic acid and fluoride ion were found in urine, blood, and saliva [65]. FR thus released interacts further with different biological systems, supporting the redox cycle of OS. Moreover, the critically ill polytrauma has a high level of OS in the time of surgery, thus increasing the redox activity (Figure 3).

Karabıyık et al. have studied the possible genotoxic effects of volatile anesthetics in patients who required general anesthesia. They studied two groups in which the general anesthesia was performed with isoflurane and sevoflurane, respectively. In both cases, they have detected changes in the DNA structure and function [66]. In a similar study conducted by Alleva et al., significant changes induced by sevoflurane on the structural integrity of DNA were reported. Moreover, they have observed a decrease in the concentration of GSH and an increased lymphocyte concentration in the first 24 hours postoperatively. This was correlated with a decrease in the body's antioxidant capacity, mostly induced by the proinflammatory activity of the general anesthetics used [67]. However, at the opposite side, Szyfter et al., in a similar study, have not identified significant genetic changes regarding sevoflurane [68]. Jin et al. have studied the inflammatory response caused by sevoflurane in comparison with the inflammatory response caused by propofol. In order to assess the inflammatory status, they have monitored a series of specific biomarkers such as TNF-alpha, IL-6, and IL-10. Following their study, an increase of the systemic inflammatory response in critically ill patients who have received sevoflurane [69] was reported. Türkan et al. have conducted a similar study in which they have quantified the OS specific for erythrocytes in the case where general anesthesia was performed with sevoflurane and desflurane. Following their study, it was reported that sevoflurane has a greater impact on the cellular redox status in comparison with desflurane [70]. Zhang et al. have studied the effects of isoflurane and sevoflurane regarding the inflammatory response and the activity of the nuclear factor NF-*κ*B in neuroglioma cells. Following this study, they have reported significant increases in the concentrations of IL-6 and NF-*κ*B, thus explaining the possible neurotoxic effects induced by general anesthesia [71]. NF-*κ*B increased activity, and, implicitly, the intensification of the inflammatory response induced by sevoflurane is also demonstrated by Li et al. in a similar study [72]. Liu et al. also demonstrate that sevoflurane is responsible for increasing the FR concentration and for altering the cell structure [73].

Although a number of specialized studies highlight the oxidative effects of general anesthetics, there are numerous studies that do not report significant redox changes, and even more they highlight a series of anti-inflammatory effects. Orosz et al. have studied and quantified a number of specific biomarkers for OS induced by general anesthesia, such as DNA damage or the body's antioxidant capacity. Following this study, significant changes regarding the cellular redox status were not identified [74]. Moreover, Schilling et al. have shown that sevoflurane is responsible for reducing the biosynthesis of proinflammatory molecules such as TNF-alpha, IL-8, and IL-1 beta [75]. Crozier et al. also show that the use of propofol and alfentanil decreases the serum levels of proinflammatory cytokines [76]. Shen et al. have identified the influence of isoflurane on the redox status of the heart muscle, reporting some cardioprotective effects [77]. Li et al. have examined the effects induced by isoflurane regarding inflammations in laboratory animals, observing a decrease of the proinflammatory factors in the lung tissue. They have also showed that neutrophils have a minimized capacity of penetration into the lung parenchyma, being responsible for reducing the production of proinflammatory cytokines. Following this study, the authors concluded a decrease of NF-*κ*B nuclear signals induced by isoflurane [78].

Numerous studies report the cardioprotective effects of volatile general anesthetics. They are able to dilate the coronary arteries by decreasing the influx of Ca^2+^ through the voltage-dependent calcium channels in the vascular smooth muscle. In addition, it was reported that halothane and desflurane increase the release of nitric oxide in the coronary arteries. Another protective effect attributed to volatile anesthetics is the anti-ischemic protection mechanisms through the conservation of adenosine triphosphate (ATP) [79]. During ischemia, ATP is degraded resulting in metabolites such as adenosine, hypoxanthine, inosine, and certain purine derivatives, which diffuse rapidly through the cell membrane. In numerous studies, significant metabolic changes caused by ischemia in the myocardium of laboratory animals treated with volatile anesthetics were highlighted. The mechanism by which volatile anesthetics help in maintaining the ATP reserves is not known, but most likely it involves increasing its synthesis [80, 81].

One of the main factors responsible for the mechanical postischemic dysfunction is represented by the alteration of the intracellular calcium concentration, given that both ischemia and reperfusion are inducing calcium overload. Increasing the intracellular calcium levels induced a change regarding the sensitivity of the contractile apparatus by decreasing the myofilaments sensitivity to calcium ions, thus a prolonged depression of the myocardial contractility being installed. The effects of anesthetics on the cardiovascular system are the consequence of their action on the calcium channel, where they cause inhibition of the atrioventricular node, prolongation of the atrioventricular conduction, and decreased myocardial contractility [80, 82].

**Figure 4:**
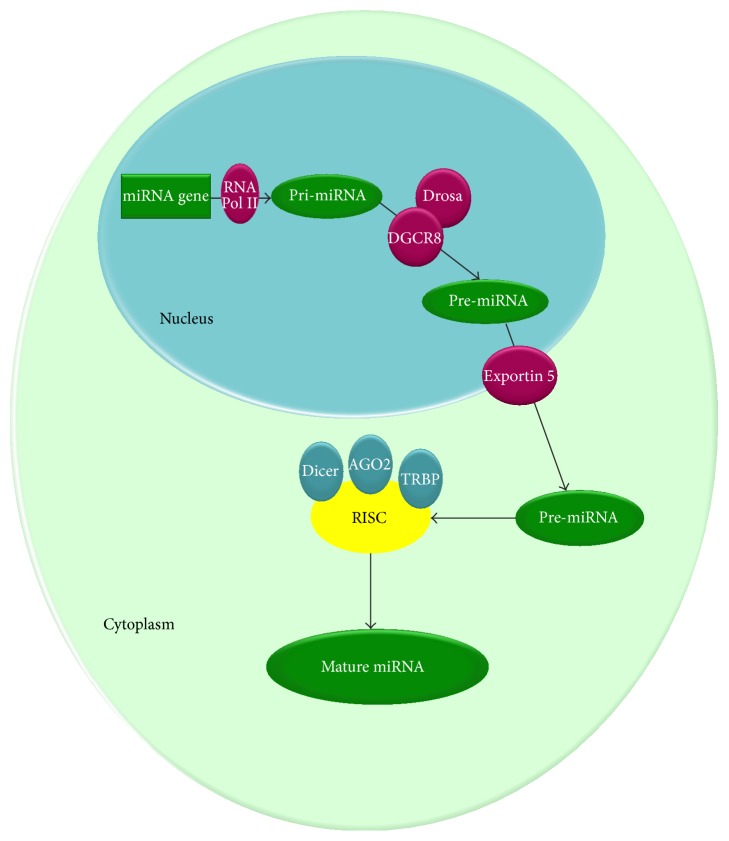
Biogenesis mechanism for miRNAs. The synthesis of miRNAs begins in the nucleus with the action of RNA polymerase II on a protein-coding. This forms a first species, called pri-miRNA. Through successive reactions of polyadenylation catalyzed by DGCR8 and Drosa, the precursor for the miRNAs species, called pre-miRNA, is obtained. pre-miRNA thus formed is transported into the cytoplasm through Exportin 5. In the cytoplasm, on the pre-miRNA acts the Dicer complex. Subsequently through the action of TRBP, AGO2 and Dicer is obtained the RNA induced silencing complex (RISC) and finnaly the mature miRNAs.

Regarding the nuclear changes induced by general anesthetics, a series of studies regarding the expression of miRNAs [83] were performed. From a structural point of view, miRNAs are short RNA species composed of 19 to 24 nucleotides on average. miRNAs genesis begins in the nucleus with the action of RNA polymerase II on a protein-coding. This forms a first species, called pri-miRNA. Through successive reactions of polyadenylation, the precursor of miRNAs, called pre-miRNA, is obtained. The new formed pre-miRNA is transported into the cytoplasm through Exportin 5. In the cytoplasm, on the pre-miRNA, the Dicer complex acts forming double stranded mature miRNA (19–24 nucleotides) and miRNA^*∗*^(passenger strand) (Figure 4) [84–87]. Kim et al. have studied the effects induced by propofol on miRNAs expression. The results are presented in Table 2.

Tanaka et al. have also identified a number of changes in the expression of miRNAs after the administration of propofol and sevoflurane. It was reported that propofol induces modifications of the miRNAs expression in a higher percentage than sevoflurane [89]. Twaroski et al. report changes in the expression of miRNA-21 when propofol is administered [90]. Another substance commonly used in the clinical practice in order to achieve sedation and anesthesia is ketamine. This is a noncompetitive antagonist of the NMDA receptors. Ketamine metabolic pathway is similar to that of cocaine, being a produced series of FR [91, 92]. Abdel-Salam et al. have studied the effects induced by ketamine on OS and on the inflammatory response, reporting significant increases of TNF-alpha in the brain [93]. Sinner et al. show an increase in the concentration of cytoplasmic Ca^2+^ in brain and an increased incidence of neuronal apoptosis when anesthesia is performed with ketamine [94]. Another molecular change in the patients who have received anesthesia is represented by the epigenetic changes. Affecting the methylation mechanism of DNA-histone chromatin complex is excessively producing a series of proinflammatory factors. Also the nuclear transcription mechanisms and the endogenous production mechanisms of some proteins are being significantly affected [95].

## 5. Antioxidant Therapy

Numerous studies present the molecular modifications induced by excessive free radicals. The physiological and biochemical modifications induced by free radicals are represented by an energetic mitochondrial imbalance, as well as a disrupted antioxidant capacity.

Lately, there have been a number of studies on minimizing the OS in critically ill polytrauma patients [15, 96]. A number of substances with high antioxidant activity were intensively studied, which include vitamin C, vitamin E, and N-acetylcysteine. Given the biological and biochemical changes induced by the direct trauma, the posttraumatic secondary injuries, and general anesthesia, implementing an antioxidant therapy is necessary in order to minimize OS. Nathens et al. have studied the effects of antioxidant therapy in the critically ill patients on a series of clinical aspects. The antioxidant therapy applied by them consisted in vitamin C 1000 mg three times per day and 1000 IU of vitamin E three times per day. Following this study, they have observed a decrease in morbidity and mortality, a lower incidence of MODS, a decrease of pulmonary complications, and implicitly a decrease of the ventilation time [53]. Crimi et al. have also studied the effects of antioxidant therapy on the critical patient. They have administered 500 mg/day of vitamin C and 400 IU/day of vitamin E. Following their study, a decrease in mortality and in the ventilation time was highlighted [97]. Collier et al. conducted a similar study in which they have reported a decrease in the length of stay in ICU and in the hospital, as well as a decrease in mortality. The antioxidant therapy implemented by them was composed of vitamin C 1000 mg three times per day, 1000 IU of vitamin E three times per day, and 200 *μ*g of selenium [98]. Fukushima and Yamazaki confirm the antioxidant and anti-inflammatory effects of vitamin C administered shortly postsurgically in critically ill patients with multiple traumas [99].

Another substance with high antioxidant capacity commonly used in clinical practice is N-acetylcysteine. Specialized studies confirm the antioxidant implications of N-acetylcysteine in the critically ill and its effects of minimizing OS and the inflammatory response. Saad et al. in a study regarding the antioxidant action of N-acetylcysteine confirm a decrease of OS along with the implementation of N-acetylcysteine antioxidant therapy [100]. Inci et al. in a similar study, but performed on patients with lung transplant, confirm that the administration of N-acetylcysteine protects the lung from ischemia through reperfusion and thus minimizes the systemic inflammatory response and reduces the intensity of OS [101]. In the specialized literature, there are a number of other studies regarding the antioxidant therapy applied in the critically ill polytrauma patients [102, 103]. Optimizing the dosage and choosing the type of substance for each type of pathology still need to be researched.

## 6. Conclusions

The biochemical implications of general anesthetics on the cellular redox mechanisms are somewhat contradictory. There are studies that emphasize the oxidative effects of anesthetic agents and a number of studies that report antioxidative characteristics. Since general anesthesia is necessary for the appropriate therapeutic management of the critically ill polytrauma patient, more intensive research is required regarding the general anesthetics redox activity. Also, a more complex research regarding the optimization of antioxidant therapy is required.

However, following the study conducted, we can affirm that the administration of substances with antioxidant capacity to the critically ill patients with multiple traumas can make great improvements to their clinical evolution.

Other studies on the molecular modifications induced by anesthesia regarding the oxidative stress in critically ill patients are required. Also, studies regarding the antioxidant therapy in patients who receive anesthesia are required.

## Figures and Tables

**Table 1:** The most important endogenous antioxidant systems.

Antioxidant system	Properties	Reference(s)
GSH	It is found in the extracellular environment as well as in the intracellular one It forms disulfide bonds with other compounds The redox activity is due mainly to the cysteine that it contains Physiological ratio of the reduced form and oxidized form in the cytosol is 50 : 1 and in the endoplasmic reticulum 2 : 1	[11, 13, 14]

Grx	It is part of the class thiol-disulfide oxidoreductases Two forms were identified in the cytosol (Grx1) and mitochondria (Grx2) Together with Trx, it modulates the cell redox activity	[15, 16]

Trx	Redox activity occurs through the action on the disulfide bonds It intervenes in the oxidation of proteins It intervenes in reducing hydroperoxides It modulates the transcription factors It intervenes in inactivating ROS	[17–19]

SOD	It is the main endogenous antioxidant system responsible for the inactivation of superoxide anion Redox activity is made by connecting with other antioxidant enzymes, such as catalase Two forms have been identified, one intracellular Cu/ZnSOD and one mitochondrial MnSOD	[20, 21]

CAT	The main activity is the reduction of hydrogen peroxide to water and oxygen It is located predominantly in peroxisomes	[11, 22]

Prx	Reduced in general hydroperoxides 15 isoforms have been identified, from which only 6 were found in the human organism Prx1, Prx2, and Prx6 in cytosol Prx5 in mitochondria and cytosol Prx3 in mitochondria Prx4 in extracellular matrix	[22–25]

PON	Extracellular oxidative enzyme 3 forms have been identified in the human organism PON1 prevents oxidation of LDL PON2 predominantly intracellular antioxidant activity PON3 is associated with the activity of HDL	[26]

GSH: glutathione; Grx: glutaredoxins; Trx: thioredoxin; SOD: superoxide dismutase; CAT: catalase; Prx: peroxiredoxin; PON: paraoxonase; LDL: low density lipoprotein; HDL: high density lipoprotein.

**Table 2:** miRNAs expression in patients who have received propofol.

miRNAs	Expression	Reference(s)
miRNA-204, miRNA-92b, miRNA-30b, miRNA-127, miRNA-296-5p, miRNA-192, miRNA-26b, miRNA-25, miRNA-186, miRNA-191, miRNA-368, miRNA-194, miRNA-199a, miRNA-23b, miRNA-133a, miRNA-219-5p, miRNA-101, and miRNA-27a	Decreased	[88]

miRNA-29b, miRNA-216a, miRNA-190b, miRNA-let-7c, miRNA-92a, miRNA-202, miRNA-140-3p, miRNA-198, miRNA-127-5p, miRNA-424, miRNA-193a-5p, miRNA-371-5p, miRNA-107, miRNA-296-3p, miRNA-143, miRNA-143, miRNA-let-7b, miRNA-30d, miRNA-302a, miRNA-103, miRNA-206-3, miRNA-153, miRNA-181d, miRNA-370, miRNA-134, miRNA-142-5p, miRNA-185, miRNA-1-let-7e, and miRNA-208b	Increased	[88]
